# Critical perspectives on rehabilitation education, practice and process: northern Honduras case study

**DOI:** 10.1186/s12913-022-08875-6

**Published:** 2022-12-21

**Authors:** Valerie Umaefulam, Isabel Cristina Gómez-Díaz, Laura Marcela Uribe-Calderón, Eliany Pedrozo-Araque, Kalyani Premkumar, Ethel Maldonado-Molina, Maria Laura Basualdo, Julia Bidonde

**Affiliations:** 1 Department of Community Health and Epidemiology, College of Medicine, University of Saskatchewan, Saskatoon, SK Canada; 2 Universidad de Santander, Facultad de Ciencias Médicas y de la Salud, Neurotrauma Centre, Bucaramanga, Santander Colombia; 3 Universidad de Santander, Facultad de Ciencias Médicas y de la Salud, Bucaramanga, Santander Colombia; 4 Universidad Nacional Autónoma de Honduras, Departamento de Rehabilitación, Facultad de Ciencias Médicas, Tegucigalpa, M.D.C. Honduras; 5 Ontario Public Interest Research Group, University of Ottawa, Ottawa, ON Canada; 6 Norwegian Institute of Public Health, Postboks 222 Skøyen, 0213, Oslo, Norway; 7 School of Rehabilitation Science, College of Medicine, University of Saskatchewan, Suite 3400, 3Rd Floor, 104 Clinic Pl, Saskatoon, SK S7N 2Z4 Canada

**Keywords:** Rehabilitation, Northern Honduras, Rehabilitation care, Rehabilitation education

## Abstract

**Background:**

Rehabilitation services are an integral part of patient care, but in many developing countries, they are not prioritized and either unavailable or easily accessible to those who need them. Although the need for rehabilitation services is increasing in Honduras, rehabilitation workers are not included in the health care model that guides the care provided to communities, particularly in rural and remote areas. To understand the need for providing impactful rehabilitation services in disadvantaged communities, we explored the education and perception of the community relating to rehabilitation, investigated training available for rehabilitation workers, and examined the rehabilitation processes and practices in Northern Honduras from stakeholders’ experiences.

**Methods:**

We utilized a qualitative descriptive and interpretive approach grounded in case study methodology to understand rehabilitation education, process, and practice in Northern Honduras. Three rehabilitation centres were purposefully selected as the cases, and participants consisted of rehabilitation workers and managers from these centres. We collected data via interviews and focus group sessions. We analyzed the data via thematic analysis using NVivo version 12.

**Results:**

In Northern Honduras, rehabilitation workers' limited training and continuing education, along with awareness about rehabilitation by community members and other health providers influence rehabilitation care. Although policies and initiatives to support people with disabilities and the broader community in need of rehabilitation exist, most policies are not applied in practice. The sustainability of rehabilitation services, which is rooted in charity, is challenged by the small range of funding opportunities strongly affecting rehabilitation care processes and clinical practices. The lack of trust and awareness from the medical profession towards rehabilitation workers sets a major barrier to referrals, interdisciplinary work, and quality of life for individuals in need of rehabilitation.

**Conclusion:**

This study advances knowledge of the need to increase understanding of rehabilitation care among community members and health providers, improve care processes and resources, and foster interprofessional practice, to enhance the quality of care and promote equitable care delivery, especially in rural and remote communities.

**Supplementary Information:**

The online version contains supplementary material available at 10.1186/s12913-022-08875-6.

## Background

Globally, an estimated 1 in 3 people today live with a health condition that can benefit from rehabilitation services [1]. Rehabilitation services are vital for patient care, but they are often not readily available and accessible to individuals who need them in many low and middle-income countries (LMIC). Many countries cannot respond to existing rehabilitation needs, let alone the forecasted increase in need [2], highlighting the requirement for increased investment in the health workforce, including rehabilitation workers. Research suggests there will be a projected shortfall of 18 million health providers by 2030, primarily in LMICs [3]. In some LMICs, more than 50% of people do not receive the rehabilitation services they require [4], which can lead to the worsening of medical conditions, new or further complications, and lifelong consequences.

Many people require rehabilitation services, but these issues are particularly challenging for people with disabilities (PWD). Disability prevalence is rising due to health and demographic trends [5]. The way societies view and understand disability affects the position of people in need of rehabilitation and their rehabilitation opportunities within the social context. In addition, some LMICs do not prioritize interactions between people in need of rehabilitation and the society in which they live.

## Honduras and healthcare

Honduras, an LMIC in Central America, has a population of 9.9 million people and a life expectancy at birth of m/f 67/73 years [6]. The country is divided into 18 departments (states) and 298 municipalities. Honduras has a two-tiered Health System with public providers, which includes the Ministry of Health and the Honduras Social Security Institute, as well as private providers [6]. According to the National Statistics Institute, in 2006, the coverage of health services was provided by: the Secretary of State (60%), Social Security Institute (15%), private sector (10%) (i.e., who have the income to pay for the services), and no access to health services (15%) [7]. The National Health Plan (2006–2010) proposed a gradual reform to the system to align with the United Nations millennium development goals [7]. In Honduras, health workers distribution in 2013 was 10.0 physicians, 3.8 nurses, and 0.3 dentists per 1,000 population [8]. The National Health Model, approved in 2013, emphasized primary health care [8]. The model guided the implementation of 500 primary healthcare teams that consists of a physician, nurse, and health promoter serving rural and hard-to-reach areas of the country. These teams prioritize economically challenged and environmentally vulnerable communities, as well as locations with situations of violence. Although there was a 6.4% prevalence of severe disability in 2013–14, the National Health Model did not include rehabilitation workers [8].

## Models of care for people with disability

Models are tools that define the basis upon which government and society can devise strategies for meeting the needs of PWD. Society’s adoption of a model (e.g., the way we see disability) can shape how organizations and our environments should be structured. The literature has discussed the medical and social models of disability to a great extent; and the charity model and human rights model (to name a few) to a lesser degree. The charity model, which is often used in Honduras, branches out from the medical model. This model has been used for fundraising and portrays PWD as those to care for or to pity. The predominant idea of the “care culture”, the institutionalization and segregation, plus the medically classification of disability and treatment, disempower people in need of rehabilitation, making them dependent and recipients of charity. Institutions and organizations, and recipients of rehabilitation donations, also follow the donors' expectations and priorities, making it impossible for them to be responsive to the real needs of people in need of rehabilitation.

On the other hand, in the social model, disability is a condition of interconnected elements perceived as related to social situations. Biological conditions are secondary to social conditions that oppress or liberate PWD [9]. The social responses to the problem should be about creating opportunities and removing barriers for the participation of PWD [10]; proponents of the social model believe the appropriate solution is the transformation of policies, laws, and public attitudes [11]. The United Nations Convention on the Rights of People with Disabilities human rights model of disability [12] offers an alternative to the criticized social model; it differs from the social model in various ways, including providing a roadmap for non-discriminatory preventative health policy and disability-inclusive development and humanitarian aid [13].

Although in Northern Honduras, the charity model is adopted to provide rehabilitation services, there is a gap in the service delivery. Despite the increasing need for rehabilitation services in Northern Honduras, there is a shortage and unequal distribution and availability of rehabilitation workers in the region. This shows the importance of understanding the factors that influence their ability to provide impactful rehabilitation services in disadvantaged communities. Our study uses a cross-disciplinary approach to offer an extended “window” into Honduran’s rehabilitation education and Honduras’ rehabilitation practice and processes. In this article, we examined the education and awareness/perception of the community regarding rehabilitation in Honduras, investigated the training available for rehabilitation workers and strategies to sustain their engagement, and explored the rehabilitation processes and practices in Northern Honduras from stakeholders’ experiences.

## Methods

### Study design

Our study utilized a qualitative descriptive and interpretative approach grounded in case study methodology [14] to understand rehabilitation education, process, and practice in Northern Honduras by exploring the perceptions of key actors and stakeholders in the region. Case study methodology allows focusing on rich cases that provide context to the study interest [15]. The consolidated criteria for reporting qualitative research (COREQ) guided the reporting of the study [16] (see Additional file 1).

The WHO global disability action plan 2014–2021 [17], WHO Rehabilitation 2030 initiative call for action [18], and Western Pacific Regional Framework on Rehabilitation [19] informed this study. The action plan addresses the goal to strengthen and extend rehabilitation and support services and community-based rehabilitation [17], while the WHO Rehabilitation 2030 call for action highlights the need to develop a strong multidisciplinary rehabilitation workforce [18] that meets the specific needs of each country. The Western Pacific Regional Framework on Rehabilitation promotes the sharing of knowledge and experiences related to rehabilitation [19]. The framework constructs related to service rehabilitation availability and quality, governance and financing, and workforce were integrated into the content of the data collection instruments.

Our study uses the WHO definition of rehabilitation: *“a set of interventions designed to optimize functioning and reduce disability in individuals with health conditions in interaction with their environment.” *https://www.who.int/news-room/fact-sheets/detail/rehabilitation

### Research team

The team consisted of bilingual (MLB, JB) and Native Spanish (EP, IC, LMUC, EM) and Anglophone (VO, KP) researchers, rehabilitation clinicians, and community members from Honduras, Colombia, and Canada. The authors are all female health professionals and researchers. VU, JB, MLB, and KP are experienced qualitative and health service researchers with expertise in research methods and community involvement. IC is a physiotherapist, LMUC is a speech therapist, and EP is an occupational therapist, and EM is a functional therapist, physical therapist, and Honduran rehabilitation academic. They all are front line and academic rehabilitation workers in Latin America.

### Data collection

To obtain an in-depth appreciation of rehabilitation, we included individuals knowledgeable about rehabilitation in the region in the study. We purposefully selected three rehabilitation centres in Northern Honduras as the cases. We chose these centres because due to our pre-existing collaboration with the centres. Some members of this team had collaborated with local organizations and health providers and conducted training workshops there to advance system changes and advance rehabilitation care [20]. As such, participants were purposefully selected and consisted of rehabilitation workers and managers of rehabilitation centres in Northern Honduras. Recruitment was facilitated via written formats such as letters of invitation and word-to-mouth invitations (i.e., snowballing). Rehabilitation workers in the context of our study refers to healthcare providers involved in providing care to people in need of rehabilitation and/or PWD. This includes physical, occupational, and speech therapists, nurses, psychologists, prosthetics technicians, and physiatrists.

We developed semi-structured interview and focus group guides (Additional file 2) for individual interviews and focus groups for each respective site. The team reviewed the guides, but they were not pilot-tested. We conducted four semi-structured interviews with three managers of the rehabilitation centres and three focus group sessions with rehabilitation workers. The focus group discussions had an average of four participants. The focus groups occurred in 2018 and 2019, while the interviews took place in 2019 and 2020. Each interview and focus group session lasted approximately 60 min and was held in person or via phone interview, whereas all focus groups occurred in person at the rehabilitation centres. Only the participants and researchers were present during data collection. A native Honduran research assistant conducted the focus group discussions and three interviews in Spanish. Another research assistant who was familiar with the rehabilitation centers and involved in providing rehabilitation workshops [20] conducted the fourth interview. The researchers explained the reasons for the research before conducting the interviews. All discussions were audio-recorded and transcribed verbatim, then translated to English or Spanish by bilingual members of the team; therefore, data were available in both Spanish and English versions for analysis.

### Data analysis

**Fig. 1:**
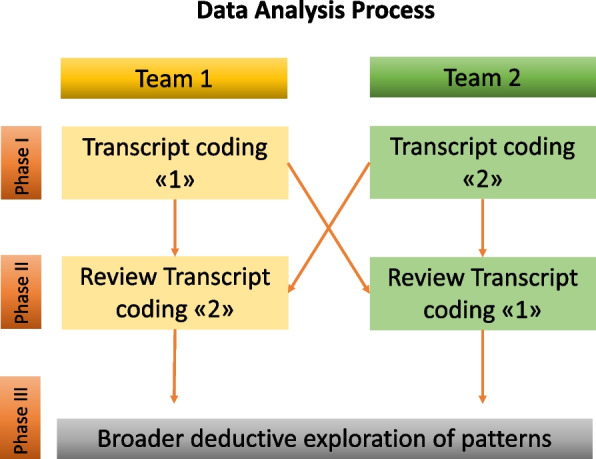
Data Analysis Process

Data analysis was managed through NVivo version 12 based on thematic analysis stages of; data immersion, initial coding, theme/category creation, and reviewing and refining themes [21]. We analyzed the data in two teams and three phases (Fig. 1); this included inductive thematic analysis by each team, a cross-team thematic analysis, and a broader, deductive exploration of patterns pertaining to specific themes of interest. Each of the two analysis teams, formed by four Anglophone and four Spanish-speaking individuals, reviewed and coded the transcripts. Teams 1 and 2 coded transcripts independently of each other. Afterward, a second team reviewed and discussed codes to ensure data congruency. We only included factors and processes supported by the data (as documented in the evidence) and if they related to the initial study propositions and research aim. The entire research team discussed emerging themes, patterns, and case outliers. Two members (VU and JB) collated and generated the themes from each team, and the entire analysis team reviewed and refined the themes.

### Reflexivity

Reflexivity is a way to identify, probe, and communicate the influence of personal perspectives on qualitative research decision-making. The team’s expertise in rehabilitation, education, and LMICs was essential for this project and provided the forum for critical reflection. All team members are affiliates of a rehabilitation network in Honduras. We used several techniques to examine our position in the research. First, we divided the team and crossed examined the data ensuring the composition of the teams were mixed (academic/rehabilitation provider). Following (or in between) examinations, we shared critical reflections on the interviews that facilitated discussion of different perspectives. We took a collaborative approach to the analysis to draw on the diverse disciplinary and interpretative skills of each team member. We discussed each team member’s relationship with the data and acknowledged its role in guiding research decisions.

## Results

### Participant’s characteristics and demographics

We recruited sixteen participants to reach content saturation. Participants were both male and female, with varying years of experience ranging from one to nine years, involved in rehabilitation services in different capacities and have been broadly classified into two categories, i.e., as a manager of a rehabilitation facility (M) and as a rehabilitation worker (RW). Table 1 shows an overview of the demographics of participants.

**Table 1:** Demographics and Characteristics of Participants

Demographics		Number
Gender	Male	2
	Female	14
Language of Communication	Spanish	14
	Bilingual	2
Roles	Manager (M)	4
	Rehabilitation Worker (RW) *Physical Therapist: 5* *Nurse: 1* *Rehabilitation Assistant: 1* *Special Ed Teacher: 5*	12

In summary and as described further in detail, participants across sites shared similar perceptions of rehabilitation in Northern Honduras despite their involvement/role in rehabilitation services. We present their perceptions under three major themes: education, process, and practice. Table 2 provides a summary of the themes and sub-themes derived from the data.

**Table 2:** Summary of Themes, Sub-themes, and Interpretation

Themes	Sub-Themes	Interpretation
*Education*	Awareness/perception about rehabilitation and disability	Knowledge of the rehabilitation organization by the general population, population perception of rehabilitation, information/awareness provided to the community, training of parents and family members, and educational, stigma, social inclusion, support programs for people with disabilities, knowledge of the rehabilitation profession, and community awareness of people with disabilities
	Rehabilitation training and continuing education	Sustained engagement of rehabilitation workers, training needs (undergraduate/post-secondary education and continuing education), poor access to training and networking opportunities, awareness and openness to learning, continuing education, and peer learning
*Process*	Rehabilitation model	Institutional funds, voluntary support, government role, and politics and support (propaganda)
	Policies	Support from social insurance, policies and support from the government (lack of regulatory policies on the profession, lack of inclusive policies, social support for the educational program), and non-application of policies (lack of application of policies)
	Access to services	Administrative process, hierarchical referral process centered on the doctor’s decision, availability of infrastructure, lack of rehabilitation workers, and geographical distribution of rehabilitation centres. Challenges and obstacles for people in need of rehabilitation
*Practice*	Clinical practice	Clinical rehabilitation services, quality and scope of services (geographic scope and number of patients covered), and infrastructure and trained providers of services (lack of infrastructure and trained human resources (accredited professionals) Lack of training of patients’ relatives/caregivers
	Inter-professional practice	Interdisciplinary teams, interaction between physicians and rehabilitation workers, Interprofessional relationships and professional collaboration, and hierarchical structure within interprofessional relations

### Education

Education as a broad theme in our study context relates to the perception of rehabilitation as a profession by the community and other healthcare professionals (i.e., medical doctors [MD]). Also, education relates to limited knowledge of rehabilitation organizations by the general population, lack of community awareness of people in need of rehabilitation, the information provided to the community, training of parents and family members, stigma, social inclusion, and support programs for people in need of rehabilitation. Education also delves into formal education training needs for health professionals, named academic programs (technicians, bachelor, postgraduate), continuing education for rehabilitation workers, peer learning, and strategies to sustain their ongoing learning engagement. There was mention of within-country educational inequalities associated with the geographical distribution of educational institutions. This represents a missed opportunity to focus on human capital and efforts to have widespread benefits in rehabilitation for Northern Honduras communities.

#### Awareness/perception about rehabilitation and disability

The importance of rehabilitation awareness and the establishment of role clarity was echoed by all participants. They described that there was a need for the general population and other health professionals, particularly medical doctors (MD)s, to be educated on the works of rehabilitation organizations and services, this is because, *“the knowledge that the community has is very little (RW).”* A participant noted,

“*There are many people in [city]who do not know who we are, taxi drivers, people who ask us where we work? We give them the location and they tell us they have never been there, or sometimes people arrive who tell us they did not know that this organization existed (RW).”*

Managers of rehabilitation centers reflected on the complexity of navigating referrals to medical doctors due to a lack of awareness of the role and function of the rehabilitation profession. As others in the network of rehabilitation care (e.g., patients, caregivers, other health care providers, and administrators) became more aware of the rehabilitation profession, the role clarity (and recognition of the profession) was gradually established. Participants recognized the value of organizational support and the discourse from all participants showed a shared agreement on the need for awareness and knowledge of rehabilitation services by physicians. Medical doctors were portrayed as having minimal to no understanding of what rehabilitation services entail. Unfortunately, physicians' lack of knowledge or understanding presently undermines the rehabilitation healthcare profession and does not actively demonstrate acceptance and integration of rehabilitation within healthcare services, impacting the lives of those with disabilities and their families. In the words of a participant,

“I feel that there is a lack of knowledge about the rehabilitation area and I think we have to work in this aspect, with doctors (M).”

A key characteristic of the rehabilitation worker-physician relationship is trust and knowledge of each other's skills. There is considerable setting-dependent variability in the number of physicians that work with the rehabilitation centres in Northern Honduras. At this stage, the development of trust is influenced by the physician’s understanding that the rehabilitation worker is knowledgeable and critical to helping the individual’s rehabilitation process. In addition, participants identified that rehabilitation workers were more likely to work in isolation and disconnected from other health professionals. Despite the perceived value of working collaboratively with physicians and rehabilitation workers to make prompt care decisions, participants noted that discrimination and hierarchical structure within the health profession relations limits care. Case in point, a participant stated,

*“Physical therapy is not well perceived by doctors, they think we are massage therapists and occupational therapists, who play with patients and put them to make crafts. When that ideology comes from above, and it becomes a culture problem, and also an education problem (RW).”*

The information and awareness provided to the community on rehabilitation workers’ role and their tasks within the disability field could impact the population’s perception of rehabilitation. In line with this thought, a participant noted,

*“we want to execute as an institution by offering workshop, by providing education, there is included what is the part of inclusion, what is the part of sensitization and promotion of this type of activities for the population in general to remove that stigma, remove that desensitization from seeing patients, people with disabilities or with a condition that they are something rare that they are not ordinary or true people (M).”*

For PWD, socially related practices such as support systems, stigma, and social inclusion influence rehabilitation practice in Northern Honduras. The stigma associated with disability affects rehabilitation practice, as stated by a participant, *“people with disabilities are discriminated against and I think that has a lot to do with the culture (M.)”* Another participant explained further that,

*“culture affects non-inclusion, because when the population does not have an education in relation to people with disabilities, either a disability or a condition, there is a certain grade of stigmatization towards that person depending on the condition or type of condition, whether intellectual, or the condition of the person (M).”*

Nonetheless, educational programs for careers of people in need of rehabilitation often incorporate, “*inclusion workshops, teacher training, teachers from different public and private schools give trainings, talks and schools for parents (M).”*

#### Training and continuing education for rehabilitation workers

All participants articulated training needs and gaps to, *“at least knowing more about the treatments that are given to patients (RW)”,* which will influence the sustained engagement of rehabilitation workers. Rehabilitation workers are willing to learn and improve skills,

*“we have to work more on trying to get more workshops... because that helps the employees too, they want to participate and they want to know, they want to get stimulation and help (M).”*

Furthermore, it was expressed that providing access to peer learning, training, and networking opportunities for rehabilitation workers could also increase engagement. As noted by a participant, *“if supervision and collaboration we can make videoconferences right? one day, one hour, once a month, to start forming what is the network of rehabilitators (M).”* Another participant mentioned the merit of peer learning via which rehabilitation workers are,

“*able to exchange and present those cases that are difficult or between themselves they also support each other and if we have that evidence based on practice…, they meet to analyze the difficult cases they have and how to help this type of people (M).”*

In all settings, the rehabilitation worker’s enthusiasm, self-organization, and role awareness enables the rehabilitation provider to either change perceptions or find strategies to maximize efficiencies.

### Process

In our study’s context, process as a theme speaks to activities, actions, service delivery models, and developments that influence rehabilitation care in the country, such as access to services and the practice of associating disability with need or misfortune (i.e., charity model).

#### Rehabilitation model

The rehabilitation centre managers emphasized the impact of policies on the process of rehabilitation care in Honduras. For example, support from social insurance was mentioned to be limited and, in some instances, non-existent. A participant stated,

“*It is called Social Security, but not everybody has access to it. It is mostly for the people who work for the government or private institutions that they pay a fee to be part of this social security (M).”*

Across all settings, funding was consistently identified as a challenge. Rehabilitation service provision in Northern Honduras is rooted in charity, which relates to how the institutions fund themselves and provide services. It also speaks of the cultural views and values Honduras as a society place on individuals with disabilities. In line with this charitable way of providing rehabilitation services, funding comes from multiple sources, including annual efforts from local/national fundraising activities, international donors, or directly from individuals’ fees. The challenge of these different sources is the dependency on intermittent short-stream funding and its impact on the centers and health professionals’ future and sustainability. In general, there is no single clear funding source but a multitude of donations and fundraising activities sustaining the rehabilitation centres. This funding approach perpetuates the practice of aiding the needy/disabled ingrained in the country and culture.

The managers of rehabilitation centres indicated that their position(s) (e.g., as board member(s)) were predominantly established by voluntary support from individuals. For instance, a participant indicated, *“this is voluntary work, so sometimes we work approximately 10 h and more, we also take care of activities so that the center works (M).”* Community fundraising events provide funding for the centre. A participant voiced, *“basically, we sustain ourselves with the marathon, and with the contribution of the companies and the socially responsible people and institutions (M).”* Another participant mentioned other activities conducted to raise funds,

*“We focus on making raffles, selling meals, we organize bingos. In some of the activities we have to use some money, for example, when there is bingo we give gifts, we go to the commercial houses who donate us gifts for bingo activities, the bingo practically leaves us a profit of approximately 50,000.00. The roasted meats that we cook add more work, we manage to sell them, but it is a very exhausting job, and we are volunteers who all work, we are all very busy, but we dedicate part of our time to carry out these activities in order to support the center (M).”*

Additionally, voluntary donations from service users at the centres contribute to the financial resource,

*“No fee is charged, what we have in the different rooms… are urns with keys so that people voluntarily contribute what they can contribute and whoever does not, is not charged anything (M).”*

Furthermore, participants indicated that there was limited support from the government. As stated, *“We are providing a service to all, right? The only true requirement is the doctor's reference. We do not have support from the government or from any other institution (M).”*

A clear example of rehabilitation centres’ neediness and helplessness is the dependence on charitable support propagandized by aspiring political leaders who make promises to support rehabilitation services during campaigns but often do not follow through, thereby impacting the care. As articulated by a participant,

*“Well politically, they go to the centers, they look for a person who needs a chair, a crutch and they give it and take photos and this and that but the support itself that this person need, he/she does not have it; I could point out that I do this and that, but the community does not receive the support as it should be (M)”.*

#### Access to services

Access to services was impacted by various factors, including administrative process, infrastructure, number and quality of rehabilitation workers, the geographical distribution of rehabilitation centres, and challenges and obstacles for people in need of rehabilitation. Participants indicated there were no administrative barriers, especially regarding referral to centres. Most centres have chosen to operate an open-door policy due to inadequate physician-rehabilitation provider collaboration, as stated above. For instance, a participant noted,

“*They have to bring a copy of their ID, they have to bring a referral from a doctor, but that is not inconvenient because everybody gets it. And, for getting the appointment, they do not need to come over here, they just call us (M).”*

A referral is essential to receiving services, but this process is centered on the doctor’s decision (their knowledge and beliefs in the rehabilitation profession), *“The referral is made by any doctor, we do not have a specific doctor, but there are several doctors who are referring patients (M).”* However, “*even though these patients do not have a referral, they are always looked after and some of these patients go because they tell them to go to the centre (M).”* Also, there is often *“a waiting list, like for one week (M)”* due to the increasing demand for rehabilitation services.

The geographical distribution of the rehabilitation centres limits service access since, *“Rehabilitation services for people with disabilities are…not… distributed evenly in Honduras (M).”* This results in inequity in the provision of rehabilitation services, subsequently causing permanent disabilities, “b*ecause they did not have a place where they could receive therapy during their first 48 to 72 h (RW).”* Another participant conveyed, *“The distances to reach the rehabilitation center are very long and usually, these people are mostly poor and have difficulty moving from their places to the center of rehabilitation (M).”* Nonetheless, although the centre's aim was to primarily serve people with very limited economic resources, due to access issues, the centres now provide services to people of all social status.

The need for both human and physical resources was a repeated mutual opinion. Participants remarked that there was limited availability of rehabilitation workers at the centres (and in the northern parts of the country in particular), thus influencing access to care. A participant stated, *“very scarce (trained professionals), and here in previous years we had to resort and look for physiotherapists in [country] (M).”*

Transportation and other disability-related challenges were equally regarded as a social factor that impacts rehabilitation practices, particularly as it relates to mobility. Regarding this, a participant conveyed that with limitations in ramp accesses at buildings and buses,

*“There is no way that these people can mobilize and it is difficult to think that a bus has access so that a person in a wheelchair or a person who does not have his vision can walk because there is no way to that … they can leave their homes (M).”*

Also, limitations in the availability of healthcare infrastructures, such as wheelchairs and hospital equipment, create a barrier to access. To emphasize this point, a participant stated, *“that limited us, that we did not have materials, ….as I said, maybe the limitations could also be the equipment in some centers (RW).”* Another participant stated, *“… we have quadriplegic patients that it is extremely difficult to mobilize, and they have to take them in an uncomfortable car (M).”*

### Practices

In our study, practices refer to exercises and procedures that impact the provision of rehabilitation care. Practice-related sub-themes included clinical, social, and inter-professional practices in rehabilitation. The scope of services provided by the centres varied and were stated to be extensive and cover a range of services by some and was considered limited by others. The reach and number of service users was commented to be extensive; however these remarks are not reflective of ongoing practices based on observations from our fieldwork in the region, *“the amount we serve is great so if you came in the morning, you would see children, you could see people at the gym (RW).”* In some settings, other non-clinical services are also provided,

“*I mean, that the organization always tries to ensure that the child is always fed, we always worry that the child is fed so that we can provide rehabilitation, we give them medicines, diapers (RW).”*

#### Clinical practice

Limited infrastructure, resources, and availability of trained providers impacted clinical practices. Two participants mentioned, *“the biggest limitation is the staff, the team (RS),” and “the centre has a very low number of professionals (RW).”* The downside of the lack of human resources is that patients often wait longer for rehabilitation services demonstrating multiple inefficiencies. The gap in rehabilitation workers for various services influenced practice. For instance, a participant noted, *“we would like to have a physiatrist [physical medicine and rehabilitation medical specialist], that is our dream, and a psychologist, exactly in terms of human resources that is what most urges us (M).”* Notwithstanding the limitations in human and physical resources, rehabilitation services are provided to individuals from varied locations, *“we already serve patients from several municipalities”*, and often serve *“…Approximately 40 to 50 patients every day (M).”*

Additionally, despite the limitation of rehabilitation workers, participants indicated that the quality of services provided was adequate, as stated, “*in the center we try to ensure that the quality of service is good. We try to make it most appropriate and that mothers are well-educated and comfortable with the children (RW).”* Two participants noted that they, “*provide the patient with quality care and warmth to achieve adequate physical and mental rehabilitation, free of charge (M)”,* and *“if a satisfaction survey could be done, I think that people would say that they are super satisfied with the service (M).”*

Socially related practices such as support systems such as self-support groups and the relationship between the centre and patients and families fostered care. In order to advance knowledge, parents and family members of PWD are trained at the centres to support care. As a participant stated,

*“The employees teach them how to handle the child in their home, how to do the rehabilitation and then there is a specific day where they supervise it. Show me how you did the (exercises with the) child and then they are under supervision (M).”*

#### Inter-professional practice

All participants buttressed the importance of inter-professional practice in rehabilitation, particularly the need for professional collaboration across interdisciplinary teams to further care.

For example, a participant noted,

“*the relationship has to be very close because a patient goes through the center from physical therapy to speech therapy or occupational therapy or goes to psychology, and the same patient can go through four areas or five then, it is very close. They have to have a very close relationship (M).”*

## Discussion

In summary, our study shows how rehabilitation workers' training, ongoing education, and awareness about rehabilitation by community members and other health practitioners influence rehabilitation care in Honduras. Education is closely linked with interprofessional practice due to the perception of the predominant role of physicians in society and in providing access to services, particularly as it relates to the referral process of persons who need to access rehabilitation services. In Northern Honduras, a small pool of funding for rehabilitation services, which is rooted in charity in the region, is challenged by sustainability, which subsequently affects rehabilitation care processes and clinical practices.

Our results indicated the importance of rehabilitation awareness amongst the general population and understanding and clarity of the roles of rehabilitation workers, particularly physicians. Similarly, a study in Honduras indicated that the attitude towards rehabilitation care amongst health professionals in Northern Honduras is positive, but health personnel in hospitals lack the knowledge and practices to treat people in need of rehabilitation and provide comprehensive treatment, which may explain the low rate of referral to rehabilitation services [22]. Thus, emphasizes the need to strengthen rehabilitation services via training not only among rehabilitation workers but also among hospital staff in all services to increase knowledge, prevention, and best practices [22]. Also, in alignment with the WHO Rehabilitation 2030 initiative and the Western Pacific Regional Framework on Rehabilitation that informed our study, knowing that the rehabilitation workforce is limited compared to other areas of health, it is essential to ensure workforce sustainability by clarifying the roles of the different rehabilitation actors to promote development and advance the profession [19].

As accentuated in our study, socially related practices, including stigma and lack of social inclusion, influence individuals in need of rehabilitation care obtained in Northern Honduras. For example, a study conducted in Roatan showed that how disability is perceived in Honduras is influenced by regional traditions; as such, there is low regional attendance of children with disabilities in schools [23]. Hence the need for support services to advance the training of family, caregivers, and community members on disability care to increase knowledge and enhance care, education, and inclusion.

The Honduran government has intimated support towards furthering rehabilitation care, as demonstrated in the signing of international agreements related to disability, still its policies are often irresponsive to the needs of PWD [24]. Despite the shortcomings in the operation, development, and national coverage of inclusive programs, known in Honduras as Community Based Rehabilitation [25], there is extensive value in furthering and establishing continuing education opportunities for rehabilitation workers to improve skills and increase engagement.

Our study indicated that irrespective of available data on the geographical distribution of people in need of rehabilitation in Honduras and national policies that have been enacted to advance the care of PWD, there is limited application of the policies. For example, at the governmental level, since 2002, the Honduran territory has had specific information that characterizes PWD, having entered the identification of this group in the Permanent Household Survey [26]. In addition, in 2004, the National Policy for the prevention of disability, the comprehensive care, and rehabilitation of PWD and the National Committee on Disability was adopted [25] to ensure the protection of their rights, the fulfillment of duties, and the development of social, economic, political, legal, and cultural actions, as well as to implement care programs with broad community participation and make sure efforts towards prevention, response, and rehabilitation of people in emergencies and disasters [27]. Nevertheless, despite these policies and their potential to further rehabilitation care, there is limited social support from the government for activities and programming at community levels to promote access to integrated care. A multisectoral approach is imperative to advance the WHO global disability action plan, which may include policy implementation at the different levels of government, along with support from development organizations, service providers, and academic institutions [17].

Rehabilitation services in Northern Honduras are provided via the charitable care model to advance care and promote positive community knowledge and attitude towards disability [23]. This is because the country's efforts in responding to the needs of the population through the creation of care centers are insufficient since these centres have little budget, and the care provided is often not sustainable. As such, civil society adopts actions to favor educational, rehabilitation, habilitation, and inclusive development processes for this population, which is mandatory by the constitutional act in the country [25]. According to the report from the Honduran organizations of persons with disabilities to the United Nations Committee on Economic, Social and Cultural Rights in 2016, the national health system of the country does not have protocols for care and medical personnel trained in disability, so there are no conditions of universal accessibility in the different health care sectors, which limits quality care and a multidisciplinary approach to care [28]. Likewise, access to rehabilitation services and technical aids for PWD is limited since most centers provide care privately, where a "fee is paid according to the service received" [28]. Some rehabilitation centres are run by community support via sponsorship from Non-Governmental Organizations to close the gaps in service access and delivery and to serve populations not served by government facilities.

Furthermore, access to services for people in need of rehabilitation is framed around the practice structure and the referral pathway for rehabilitation care. Referrals are mostly provided and centralized around medical doctors. Rehabilitation workers are unable to make any referrals and connect with other professionals to further patient care. This issue is not unique to Honduras. For example, Ragupathi et al. [29] noted that the most cited barrier to cardiac rehabilitation in LMIC was the lack of physician referral. Authors suggest that patients, physicians, and the system should find ways to improve access to rehabilitation. In Honduras, rehabilitation workers are dependent on the direction of the medical doctors because they make all the decisions. For instance, after client evaluations, therapists refer patients back to medical doctors to endorse the start of the rehabilitation service process. As indicated in our study, there is a growing need for quality rehabilitation service which is prompt, effective, and efficient. Due to the growing demand for rehabilitation services, referral only from medical doctors adds another layer to the access barrier, resulting in a long waiting time from the beginning of the assessment to subsequent rehabilitation process, and this practice structure also limits interprofessional practice. The European Commission 2006 report [30] suggests that a ‘spiral’ model of referral may be more appropriate. Patients should be referred within primary care and between different levels of the system on an ongoing basis. This ‘spiral’ would require a high degree of coordination, an explicit definition of the responsibilities of the providers involved and good information for patients. Furthermore, it is important to include the services of rehabilitation workers, who can act as the first point of contact in the health system along with primary care physicians and foster the strengthening and integration of rehabilitation services [31].

In addition, Honduran’s rehabilitation workers such as functional therapists have not been able to attain professional status, which is a major barrier to enabling them to take more prominent roles in patient care and provide patient referrals [32]. Given this, for more than 18 years, there has been ongoing advocacy and request to the National Congress to approv the creation of the College of Therapists of Honduras professional association; however, this request has not yet been accepted [32]. The approval of this association would facilitate the referral processes can occur from other rehabilitation workers such as psychologists, speech pathologists, and functional therapists who provide care for children and adults who have some type of cognitive, motor, or sensory disability.

Our study showed that the geographical distribution of health facilities, the limited availability of rehabilitation workers at the centres in Northern Honduras, and physical infrastructure are crucial in advancing access and rehabilitation care. A systematic review of access to rehabilitation in LMICs suggested that PWD are not receiving a range of specific health services required to improve functioning [4], which may be due to limited services and health providers in the region. As mentioned in our study, rehabilitation care in most rural communities, often served by the community-based rehabilitation program, are non-for-profit and frequently does not have access to extensive infrastructure compared to the urban areas. Most available infrastructure is in the urban areas of Honduras, leaving the rural area, such as the northern area of Honduras without support. Thus, affecting the access of people in need of rehabilitation to assessments, rehabilitation, and follow-up processes. Services provided by local rehabilitation centers that support the entire Northern Honduras population fall short in infrastructure/resources and the number of human resources available in rehabilitation, from physiotherapy, occupational therapy, and speech therapy.

As reported by Mejia et al. (2014), there are twenty-six rehabilitation centers in Honduras, and only seven of these centers have a rehabilitation specialist, highlighting the need to train rehabilitation workers since there is an unsatisfied demand due to the increase in chronic non-communicable diseases, life expectancy, and the consequences of accidents and violence in the country [33]. There has been an increase in technical training provided by universities and an increase in the number of graduates in Honduras, thereby improving the practice workforce of physical and functional technicians. However, in contextualizing this boost, trained rehabilitation workers often do not go to remote regions to provide services. As such, there is still a gap in the healthcare workforce needed in rural communities. It is essential to promote the equitable distribution of rehabilitation workers in rural communities.

Our study has highlighted the need for interprofessional practice and education. The hierarchical structure within interprofessional relations is linked with interprofessional education (IPE) and the perception of the prominent role of physicians in society. IPE is needed to respond to the changing demands of the environment, and Honduras has made some efforts/inroads in this regard, which have been led by the Pan American Health Organization and the World Health Organization (PAHO/WHO), and regional technical meetings have been held in which Honduras has participated, creating the Regional Network of Interprofessional Education of the Americas (REIP) [34]. One of the most important strategies of the REIP has been to support countries to understand better the human resources strategy for universal access to health approach and implement it through action plans [34]. Despite the participation of Honduras in furthering the integration of the different members of the team to provide better services, the hierarchy of the medical doctor’s role in relation to interprofessional relationships, perceived by the participants in our study, may be associated with the position that doctors have historically exercised in society and support that the medical profession receives compared to other health professionals. It is crucial that health systems at all levels ensure that rehabilitation is integrated into interprofessional practice to maximize access, further continuity of care, and achieve universal health coverage [4].

## Strengths and limitations of study

The multidisciplinary research team that included researchers and clinicians internationally and from the region fostered rich interpretive and analytical discussions grounded in the context of ongoing rehabilitation practices in Northern Honduras. All authors are affiliated with the same rehabilitation network as such, are biased toward rehabilitation care in Northern Honduras. Nonetheless, we utilized several strategies to ensure that the results reflected participants’ discussions and our collective experiences with rehabilitation education and practice in Northern Honduras fostered a rich data interpretation. Our study would have provided a broader perspective on rehabilitation education, practice, and process with the inclusion of the voices of service users, i.e., people in need of rehabilitation and family members. Nonetheless, we were able to report the perspectives of individuals who were fully engaged and involved in providing rehabilitation services in different capacities from various locations; thus findings are likely representative and relevant to rehabilitation care in the region.

## Conclusion

Our study in Northern Honduras highlights the gaps in rehabilitation education, processes, and practice. It is imperative to increase the understanding and awareness of rehabilitation care among community members and health providers to improve attitudes toward disability and care practices. We recommend strengthening capacities and improving quality service by furthering internal training within institutions to meet the high demands of specialized services and build the necessary competence to meet the needs of the population [35]. Community members in need of rehabilitation in Northern Honduras will benefit from enhanced care processes and resources, including referral processes, availability of infrastructure and rehabilitation workers, easily accessible across the geographical region, and improved implementation of social policies. Enhanced interaction and collaboration between physicians and rehabilitation workers will further the quality of care and promote equitable and sustainable rehabilitation care, especially in rural and remote communities.

## Supplementary Information

Below is the link to the electronic supplementary material.

Supplementary file1. COREQ checklist (PDF 453 KB)

Supplementary file2. Focus Group and Interview Guide Questions (DOCX 22 KB)

## Data Availability

The datasets used and/or analysed during the current study available from the corresponding author on reasonable request.
