# Supplementary material for: Critical perspectives on rehabilitation education, practice and process: northern Honduras case study
Source: BMC Health Serv Res. 2022 Dec 21;22:1561. doi: 10.1186/s12913-022-08875-6 (PMC9768948; doi:10.1186/s12913-022-08875-6)
Supplement: Supplementary file 2 — Supplementary file2. Focus Group and Interview Guide Questions (DOCX 22 KB) [file 12913_2022_8875_MOESM2_ESM.docx]

**Additional File 2. Focus Group and Interview Guide Questions**

**Focus Group Guide Questions**

1. Thinking back to the workshop, can you tell me a little about your experience at the workshop?  What are a few main points you recall about the workshop? (5 m)
2. I would like you now to consider the effects of the workshop your knowledge related to your work with children with disabilities.  Can you tell me about the effect of the workshop on?
   1. Your knowledge related to the conditions they have (in what ways?)
   2. Your knowledge related to assessing
   3. Your knowledge related to planning treatments
   4. Your knowledge related to treating children with disabilities
3. I would like you now to consider the effects of the workshop your skills related to your work with children with disabilities.  Can you tell me about the effect of the workshop on
   1. Your skills assessing
   2. Your skills related to treating children with disabilities
4. Thinking back, did the workshop affect your confidence in working with children?  Interacting with parents?  How did you notice this?
5. What are your thoughts on your connections with others in the area of rehab.  Did your attendance at the workshop affect this in any way? Please explain any effects.
6. Do you have other comments about this workshop?

**Interview Guide Questions**

1. Are there job opportunities specifically for people with disabilities?
2. Are there programs dedicated to the labor inclusion of people with disabilities?
3. Are there education opportunities for people with disabilities?
4. Okay let's go to rehabilitation services for people with disabilities. Would you say that rehabilitation services for people with disabilities are distributed in a uniform way in Honduras?
5. How does the department where you work with other departments compare?
6. Do you know any public policy on the disabled in Honduras?
7. How does the Government support people with disabilities in Honduras? If they have any support!
8. We will talk about referral practices and admission to the rehabilitation service. Does the health system of Honduras cover some rehabilitation service? as for example physiatry, physiotherapy or phonoaudiology; for example, most of all in insurance, social security.
9. With what professionals does the institution have to offer the rehabilitation service?
10. Are users of the rehabilitation service referred by a doctor or can they be treated directly?
11. Description of the rehabilitation centers that is the other section and this goes as in general: What is the relationship between the rehabilitation center and the government?
12. What is the autonomy of the rehabilitation service?
13. Is there an accreditation process for rehabilitation centers?
14. What are the hours of operation of the centers?
15. How many rehabilitation professionals and others work at the center?
16. How are services financed? Who pays for the rehabilitation service (patient, NGO, government, health service?)
17. If there is a commission for presenting services, can you inform me about the distribution of the rate (the rehabilitation service keeps everything or something going to the government?) How do you distribute that money you get?
18. Barriers to accessing rehabilitation services. In your opinion, what are the obstacles that people with disabilities face in receiving care?
19. Do you know if there are administrative barriers for the patient to receive rehabilitation services? for example: admission to the service
20. Practices of the rehabilitation professions in Honduras: Physiotherapy, occupational therapy and speech therapy practices are regulated by the government of Honduras?
21. How is the relationship between the rehabilitation members for example doctors, nurses, psychologists, physiotherapists, physiatrists and others? I mean if they work as a team
22. What is the profile of the rehabilitation center, of the rehabilitation department speaking generally in Honduras?
23. Is there a registration process for the therapists? For example, doctors; the doctors have their doctor's school, and they have their talks, they have their calls to meetings and all that
24. What is the perception of the population about the rehabilitation professions? For example: physiotherapy, occupational therapy, and speech therapy!
25. Is there any regulation or law on who can exercise physical therapy or physiotherapy and what is the scope of the profession in Honduras?
26. Are there formal associations for physiotherapy in Honduras?
27. Under what circumstances do you assign tasks of trained therapists to therapists without training or family members or others? For example, if you have a patient and under what circumstances do you assign a certain type of task to the family member, for example, to the mother who takes your child?
28. Would you be willing to join a team to help develop a set of criteria for assigning tasks to untrained therapists or volunteers attending future workshops?
29. Would you be willing to join a team to help develop a set of criteria, ie rules, for assigning tasks to therapists or untrained volunteers who attend future workshops? for example if you receive a workshop
30. About the compensation that rehabilitation professionals receive, can you tell me if the professional has a defined salary or receives a payment per session?
